# Transwell membrane material affects myogenic differentiation in human primary myoblast‐adipocyte co‐culture

**DOI:** 10.14814/phy2.70939

**Published:** 2026-06-08

**Authors:** Claire Plissonneau, Bjorn T. Tam, Pierre Y. Garneau, José A. Morais, Alisa Piekny, Sylvia Santosa

**Affiliations:** ^1^ Department of Health, Kinesiology, and Applied Physiology Concordia University Montréal Quebec Canada; ^2^ Metabolism, Obesity, and Nutrition Lab, School of Health Concordia University Montreal Quebec Canada; ^3^ Centre de Recherche‐Axe Maladies Chroniques, CIUSSS‐NIM Hôpital du Sacré‐Coeur de Montréal Montreal Quebec Canada; ^4^ Academy of Wellness and Human Development, Faculty of Arts and Social Sciences Hong Kong Baptist University Hong Kong SAR China; ^5^ Dr. Stephen Hui Research Centre for Physical Recreation and Wellness, Faculty of Arts and Social Sciences Hong Kong Baptist University Hong Kong SAR China; ^6^ Département Du Chirurgie Hôpital Du Sacré‐Coeur de Montréal Montréal Quebec Canada; ^7^ Division of Geriatric Medicine, Department of Medicine McGill University, McGill University Health Centre (MUHC)‐Montreal General Hospital Montreal Quebec Canada; ^8^ Department of Biology Concordia University Montreal Quebec Canada

**Keywords:** adipocyte, co‐culture, differentiation, myoblast, transwell

## Abstract

Transwell co‐culture systems are models for examining cellular crosstalk, but the impact of porous membrane material on myogenic outcomes remains uncharacterized. We investigated the influence of transwell membrane material on myoblast differentiation in co‐culture with human primary adipocytes and in varying concentrations of insulin‐transferrin‐selenium (ITS). Primary human myoblasts were differentiated on 0.4 μm polycarbonate or polyester membranes, either alone (Myo‐Only) or with adipocytes (Myo + Adipo) in two different ITS concentrations ([ITS]; 0.5× or 1×). Myogenesis was evaluated by immunofluorescence, using fusion index (%) and myotube thickness (μm), and adipokine levels were measured in each co‐culture compartment. Polycarbonate membranes supported a greater fusion index and myotube thickness than polyester at both 1× (*p* < 0.001) and 0.5× ITS (*p* = 0.002). Across [ITS] (1× *p* = 0.035; 0.5× *p* = 0.006), the fusion index was higher in Myo + Adipo co‐cultures than in Myo‐Only conditions. Leptin diffusion was reduced across polyester compared to polycarbonate membranes (0.5× *p* = 0.036; 1× *p* = 0.091), whereas adiponectin diffusion was similar between materials. These findings underscore that membrane material and medium composition are critical variables in transwell adipocyte‐myoblast co‐culture systems. This study highlights the importance in the selection and reporting of these parameters to improve reproducibility across future studies.

## 1 INTRODUCTION

Signaling between skeletal muscle and adipose tissue plays a mutual role in their growth, function, and differentiation. Adipocyte‐derived factors have been shown to alter myogenic differentiation (Park et al., 2013; Seo et al., 2019), and reduce insulin sensitivity, a process that is itself essential for proper myogenic differentiation (Dietze et al., 2002; Gong et al., 2020; Kovalik et al., 2011; Kudoh et al., 2018). Using in vitro culture systems can isolate signaling between these two cell types, offering a valuable framework for understanding cellular communication.

Transwell co‐culture systems are commonly employed to model paracrine communication in vitro (Bruckbauer & Zemel, 2011; Choi & Myung, 2014; Dietze et al., 2002; Kudoh et al., 2018; Ojima et al., 2025; Pandurangan et al., 2015; Park et al., 2013; Seo et al., 2019; Sun & Zemel, 2009). These systems physically separate cell types while permitting selective molecular diffusion (Shahin‐Shamsabadi & Selvaganapathy, 2020). Two of the most commonly used materials in transwell systems are polycarbonate and polyester. These materials differ in their physicochemical and structural properties, including pore density, surface topography, and chemical composition (Corning, 2014). For example, polycarbonate membranes generally have a higher pore density than polyester membranes, which could allow for greater diffusion of molecules. However, many models examining skeletal muscle‐adipose tissue interactions have largely overlooked these variables. While researchers have studied the biological aspects of these models (Bruckbauer & Zemel, 2011; Choi & Myung, 2014; Dietze et al., 2002; Kudoh et al., 2018; Ojima et al., 2025; Pandurangan et al., 2015; Park et al., 2013; Seo et al., 2019; Sun & Zemel, 2009), none have considered how the properties of the transwell membranes affect outcomes. Clarifying the impact of membrane materials on cellular signaling is essential to improve reproducibility and physiological relevance in transwell‐based studies.

When co‐culturing adipocytes and myoblast cells in transwell systems, one of the main challenges is reconciling their distinct media requirements. Adipocytes are usually differentiated before co‐culture with myoblasts (Song et al., 2023; Vis et al., 2020). Once combined, researchers often choose between two strategies: using a single differentiation medium (myoblast differentiation medium) for both cell types or maintaining each in its own medium within separate compartments (Song et al., 2023). While the first approach simplifies the experimental design, the approach compromises adipocyte function, highlighting the need for optimized media formulation. In the latter approach, components from each medium can diffuse across the membrane, potentially altering cell behavior and reducing reproducibility. Furthermore, the physiological relevance of this system is limited because signaling gradients inevitably form across the membrane, preventing complete isolation of the microenvironment (Vis et al., 2020).

The media for differentiated adipocytes and the induction of myoblasts are similar except for the concentration of insulin‐transferrin‐selenium (ITS) supplement (1× for adipocytes, none for myoblasts). Determining a common medium would improve reproducibility, reduce cross‐differentiation issues, and simplify the interpretation of results. A 50:50 medium (i.e., one that contains 0.5× ITS) may provide a balanced environment for both cell types while minimizing the limitations of using two different media. However, this approach may not fully meet the needs of either cell type, so pilot testing is needed to confirm viability and functionality. Therefore, the objective of this study was to examine how membrane material (polyester vs. polycarbonate) and ITS concentrations (0.5× and 1×) impact how adipocytes affect myoblast differentiation in a transwell culture system. We hypothesize that neither material nor ITS concentrations would have an effect on our outcomes.

## 2 MATERIALS AND METHODS

### 2.1 Adipocyte cell isolation and differentiation

Primary human preadipocytes were isolated and differentiated from abdominal subcutaneous adipose tissue obtained during bariatric surgery from a female donor with obesity at the Centre intégré universitaire de santé et de services sociaux du Nord‐de‐l'Île‐de‐Montréal (CIUSSS‐NIM), Montréal, QC, Canada, as previously described (Santosa et al., 2015; Tchkonia et al., 2005). This study was conducted in accordance with the Declaration of Helsinki and approved by the Comité d'éthique de la recherche du CIUSSS‐NIM (400004176) and Comité central d'éthique de la recherche du ministre de la Santé et des Services sociaux (CCER 15‐16‐19). The participant provided written informed consent prior to study participation.

**FIGURE 1:**
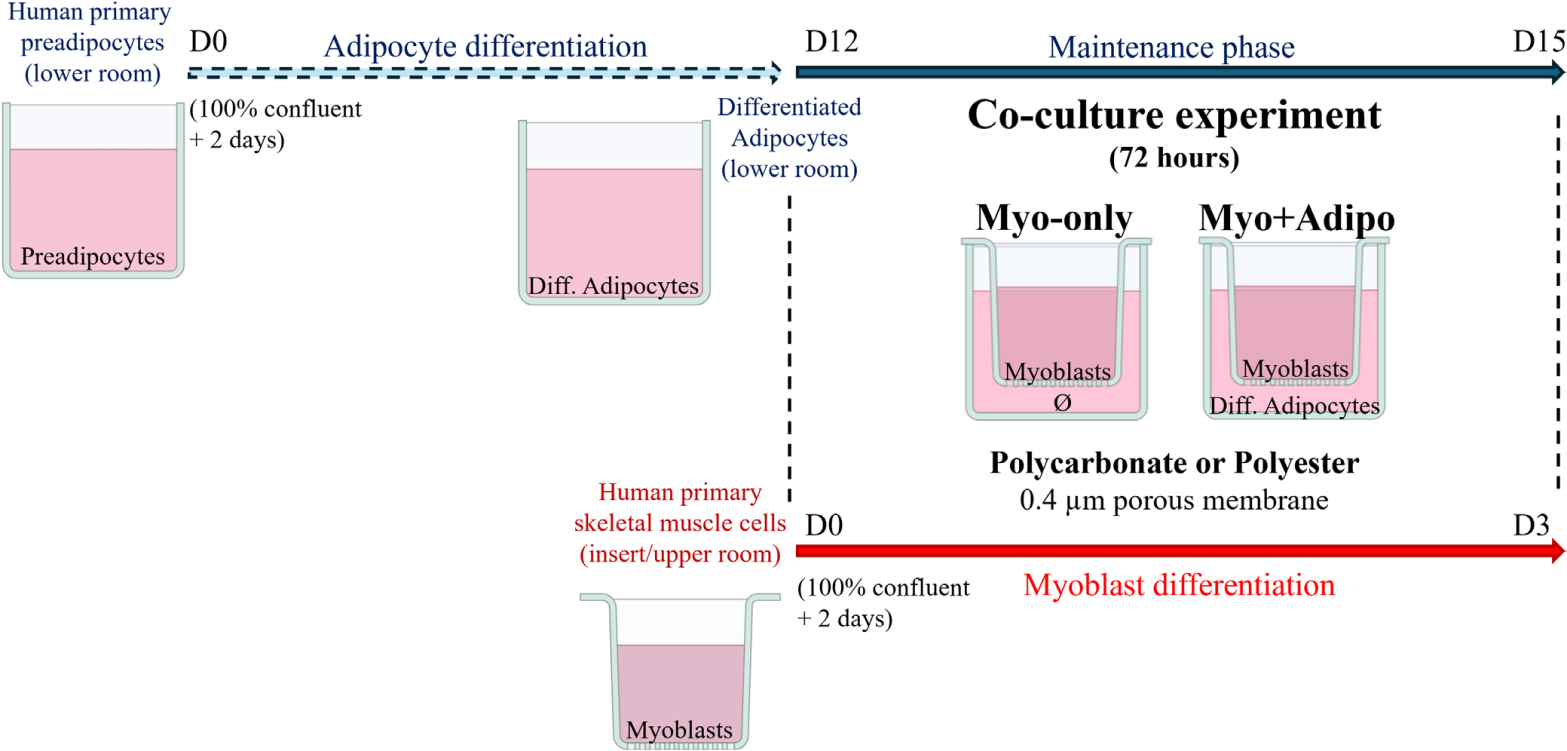
Experimental design. Human primary preadipocytes (lower room) were differentiated over 12 days. In parallel, human primary myoblasts (upper room) were seeded onto 0.4 μm polycarbonate or polyester transwell inserts coated with Matrigel. Myoblasts were differentiated alone (Myo‐Only) or in co‐culture with differentiated adipocytes in the lower compartment (Myo + Adipo) for 72 h at 37°C and 5% CO_2_.

The preadipocyte growth media was composed of αMEM (Cat#310‐010 CL, Wisent, QC, CA), 20% Cosmic Calf™ Serum (Cat#SH3008703, Cytiva HyClone™, MA, USA) and 2× Penicillin–Streptomycin (Cat#SV30010, Cytiva HyClone™, MA, USA). Briefly, primary preadipocytes were seeded in 12‐well plates (Cat#SP41123, BioBasic, ON, CA) at 1 × 10^5^ cells per well and maintained in growth medium until they reached 100% confluence. After 2 days at confluence, differentiation was induced from day 0 to day 3 using DMEM/F12 (1:1) (Cat#12634028, Gibco™, MA, USA) supplemented with 1.2 IU.mL^−1^ insulin (Humulin®; Cat#HI0210, Eli Lilly, IN, USA), 0.1 μM dexamethasone (Cat#D4902, Sigma‐Aldrich, MA, USA), 250 μM 3‐isobutyl‐1‐methylxanthine (IBMX; Cat#PHZ1124, Invitrogen, MA, USA), 33 μm biotin (Cat#B4639, Sigma‐Aldrich, MA, USA), 0.2 nM triiodothyronine (T3; Cat#16028–250, Cedarlane, ON, CA), 17 μm pantothenic acid (Cat#P5155, Sigma‐Aldrich, MA, USA), 10 μg.ml^−1^ transferrin (Cat#T8158, Sigma‐Aldrich, MA, USA), 2 μM rosiglitazone (Cat#71740, Cayman Chem, MI, USA), 20 μM indomethacin (Cat# I7378, Sigma‐Aldrich, MA, USA), and 50 μg.mL^−1^ gentamicin sulfate (Cat# GTA401.10, BioShop Canada Inc., ON, CA). From day 3 to day 12, cells were maintained in differentiation maintenance medium with the same composition but without IBMX. On day 12, differentiated adipocytes were co‐cultured with skeletal muscle cells (Figure 1). Co‐culture experiments included *n* = 6 polycarbonate and *n* = 7 polyester technical replicates.

### 2.2 Myoblast culture

Human primary myoblasts (ATCC, PCS‐950‐010, female neonate) were grown in matrigel‐coated flasks (Shahini et al., 2018) with myoblast growth media, composed of F10 Nutrient Mixture (Ham's) medium (Cat# 318‐051 CL, Wisent, QC, CA), 20% Cosmic Calf™ Serum (Cat# SH3008703, Cytiva HyClone™, MA, USA), 10 ng.mL^−1^ Epidermal Growth Factor (Cat# PHG0313, Gibco™, MA, USA), 1 μM Dexamethasone (Cat# D4902, Sigma‐Aldrich, MA, USA), 25 pmol.L^−1^ Insulin (Cat# I2643, Sigma‐Aldrich, MA, USA), 1× Antibiotic‐Antimycotic (Cat# 15240062, Gibco™, MA, USA), and 5 μg.mL^−1^ Gentamicin Sulfate (Cat# GTA401.10, BioShop Canada Inc., ON, CA). Polycarbonate (Cat# 29442–086, Corning®, NY, USA) or polyester (Cat# 29442–078, Corning®, NY, USA) transwell membrane inserts were manually coated with Matrigel™ (Cat# 354234, Corning®, NY, USA) prior to cell seeding (Shahini et al., 2018). Pore density was 1 × 10^8^ pores.cm^−2^ for the polycarbonate membranes and 1 × 10^6^ pores.cm^−2^ for the polyester membranes; however, both membrane types had the same diameter (12 mm), pore size (0.4 μm), and thickness (10 μm). Once myoblasts reached 80%–90% confluence, they were seeded at a density of 5 × 10^4^ cells per insert. After reaching full confluence, cells were maintained for an additional 48 h before the initiation of myogenic differentiation under co‐culture conditions (Figure 1).

### 2.3 Co‐culture conditions for myogenic differentiation

To compare the effects of adipocytes on myocyte differentiation, myoblasts in the transwell insert (upper compartment) were differentiated alone (Myo‐Only) or in co‐culture with differentiated adipocytes in the lower compartment (Myo + Adipo) (Figure 1). The medium was composed of DMEM/F12 (Cat#12634028, Gibco™, MA, USA) supplemented with 2% horse serum (Cat# 16050122, Gibco, NY, USA), 1% Antibiotic‐Antimycotic (Cat# 15240062, Gibco, NY, USA), 5 μg.mL^−1^ of gentamicin sulfate (Cat# GTA401.10, Bioshop Canada Inc., ON, CA), and either 0.5× or 1× ITS (Cat#41400045, Gibco, NY, USA). Co‐cultures were maintained for 72 h at 37°C and 5% CO_2_ with daily medium changes.

### 2.4 Adipokine quantification

On day 15, culture media were collected from each of the compartments of the co‐culture system (Myo + Adipo; Figure 5e). Media from the upper compartment in the co‐culture system represented the skeletal muscle fraction, whereas media from the lower compartment represented the adipocyte fraction. Leptin (Cat#DY398, R&D Systems, Minneapolis, MN, USA) and adiponectin (Cat#DY1065, R&D Systems, Minneapolis, MN, USA) levels were quantified by ELISA assay according to the manufacturer's instructions. Technical replicates consisted of *n* = 5 across all experimental conditions.

### 2.5 Immunofluorescence staining

After 72 h of incubation, skeletal muscle cells were fixed with 4% paraformaldehyde (PFA, Cat#sc‐281,692, Santa Cruz Biotechnology, TX, USA). Fixed cells were permeabilized with 0.3% Triton X‐100 in PBS (Cat#TRX506.500, Bioshop Canada Inc., ON, CA), and non‐specific binding was blocked by incubating samples with 2% bovine serum albumin (BSA, Cat#ALB001.100, Bioshop Canada Inc., ON, CA) in PBS.

Primary staining was performed overnight at 4°C using anti‐Myosin Heavy Chain (MyHC) monoclonal antibody (MF20, 1:500 dilution from the manufacturer's stock, Cat#14‐6503‐82, Thermo Fisher Scientific, MA, USA). Samples were washed with PBS, then incubated for 2 h at room temperature with Alexa Fluor 568‐conjugated goat anti‐mouse IgG secondary antibody (1:1000 dilution from the manufacturer's stock, Cat#A11004, Thermo Fisher Scientific, MA, USA). After this incubation, samples were washed with PBS, and nuclei were stained with DAPI (1:1000 dilution from the manufacturer's stock, Cat#62248, Thermo Fisher Scientific, MA, USA).

### 2.6 Immunofluorescence imaging

After immunofluorescence staining, skeletal muscle cells were imaged using a Photometrics Evolve 512 camera mounted on a Nikon Ti Microscope equipped with a 40× Plan Apo lens (N.A. = 0.95). Cells were imaged using the 89North Heliophor light engine with 405, 488, and 555 nm LEDs and appropriate filters (DAPI: 460/50 nm, GFP: 535/25 nm, Alexa568: 560/40 nm, 630/75 nm).

**FIGURE 2:**
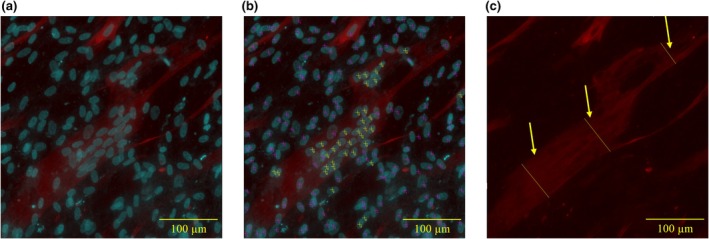
Representation of fusion index and myotube thickness measurements. Myosin Heavy Chain (red) was labeled with Alexa Fluor 568‐conjugated monoclonal MF20 antibody, and nuclei were stained with DAPI. (a) Merged image showing myotube and nuclei. (b) Fusion index (%) was calculated as the number of nuclei within the multinucleated myotubes (yellow) divided by the total number of nuclei within the field of view (purple + yellow). (c) Myotube thickness (μm) was measured by drawing three perpendicular lines (yellow) at distinct points along each multinucleated myotube and averaging the width. Images were acquired using a Photometrics Evolve 512 camera on a Nikon Ti Microscope ‐ 40× Plan Apo lens and N.A. = 0.95.

For each analysis, we acquired a total of ten 2 × 2 stitched images per well, ensuring clear visualization of nuclei and myotubes (Figure 2a). The fields of view were randomly selected using the field selection option of the microscope software (NIS Elements V5.11), minimizing sampling bias. On average, we quantified about 2000 nuclei per well (~200 nuclei per composite image).

### 2.7 Fusion index and myotube thickness measurements

Myoblast differentiation was quantified by myotube thickness and fusion. Fusion index was assessed using FIJI (ImageJ) software. The fusion index (%) was calculated (Figure 2b) using the following equation:

$$\begin{array}{c} \text{Fusion index}\;\left(\%\right)\\ \;=\frac{\text{Number of nuclei within polynucleated myotubes}}{\text{Total number of nuclei}}\times 100 \end{array}$$

Myotube thickness (μm) was measured by drawing perpendicular lines at three distinct points along each polynucleated myotube within the field (Figure 2c) to calculate the average width (μm).

### 2.8 Statistical analysis

Statistical analyses were conducted using R Studio (v2026.01.1, MA, USA). Data were tested for normality using Kolmogorov–Smirnov. Myogenic differentiation, fusion index (%), and myotube thickness (μm) were log‐transformed. Two‐way ANOVA was used to evaluate the effects of transwell membrane composition (PolyC vs. Poly E; Poly effect), ITS concentration (0.5× and 1×; [ITS] effect), and culture condition (Myo‐Only vs. Myo + Adipo; Culture effect). Tukey post‐hoc and Least Squares Means were used to examine significant interactions. *t*‐tests were used to compare the deltas of the lower‐upper compartments between materials. To help with data interpretation, values were back‐transformed to the original scale for presentation. Results are presented as mean ± SEM. Data were considered statistically significant at *p* < 0.05.

## 3 RESULTS

### 3.1 Enhanced myotube fusion and thickness with polycarbonate compared to polyester membranes

**FIGURE 3:**
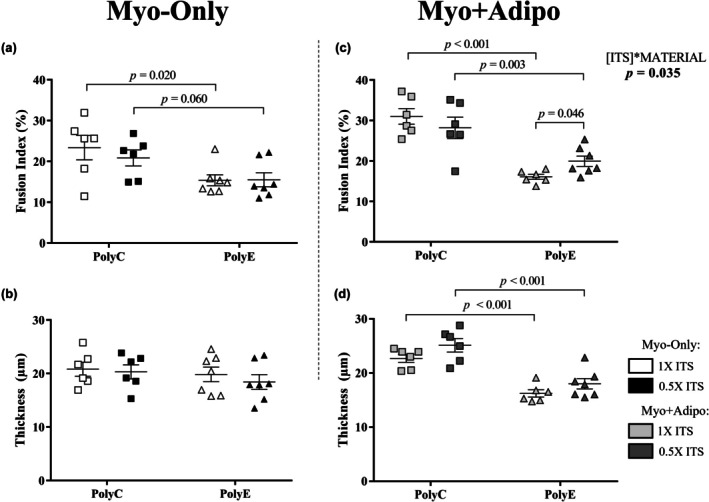
Fusion index and myotube thickness shown according to culture conditions: Myo‐Only (a, b) and Myo + Adipo (c, d). Two‐way ANOVA (log‐transformed data) was used to assess the effect of ITS concentration ([ITS] ‐ 0.5× vs. 1×) and transwell membrane material (MATERIAL ‐ PolyC vs. PolyE) within each culture condition. Fusion index is expressed as percentage (%), and myotube thickness as micrometers (μm). Data are represented as means ± SEM. Technical replicates: *n* = 6 for PolyC and *n* = 7 for PolyE. ITS, insulin‐transferrin‐selenium, PolyC, polycarbonate; PolyE, polyester.

Myoblast differentiation is characterized by the formation of elongated myotubes (Figure 2). These myotubes contain multiple nuclei and thus, differentiation is quantified through thickness and fusion. In the Myo‐Only condition, there was a main effect of membrane material (MATERIAL) on the fusion index, where the fusion index was higher (*p* = 0.004) for myoblasts cultured on PolyC than on PolyE (Figure 3a). No effect was observed on myotube thickness (Figure 3b).

When myoblasts were co‐cultured with adipocytes (Myo + Adipo), there was a MATERIAL*[ITS] interaction (*p* = 0.035) in the fusion index, indicating that the effects of [ITS] depend on the type of transwell membrane (Figure 3c). There was also an effect of MATERIAL (*p* < 0.001) with fusion index and myotube thickness higher with PolyC than PolyE (Figure 3c,d).

### 3.2 Both 0.5× and 1× ITS support adipocyte function and myoblast differentiation

**FIGURE 4:**
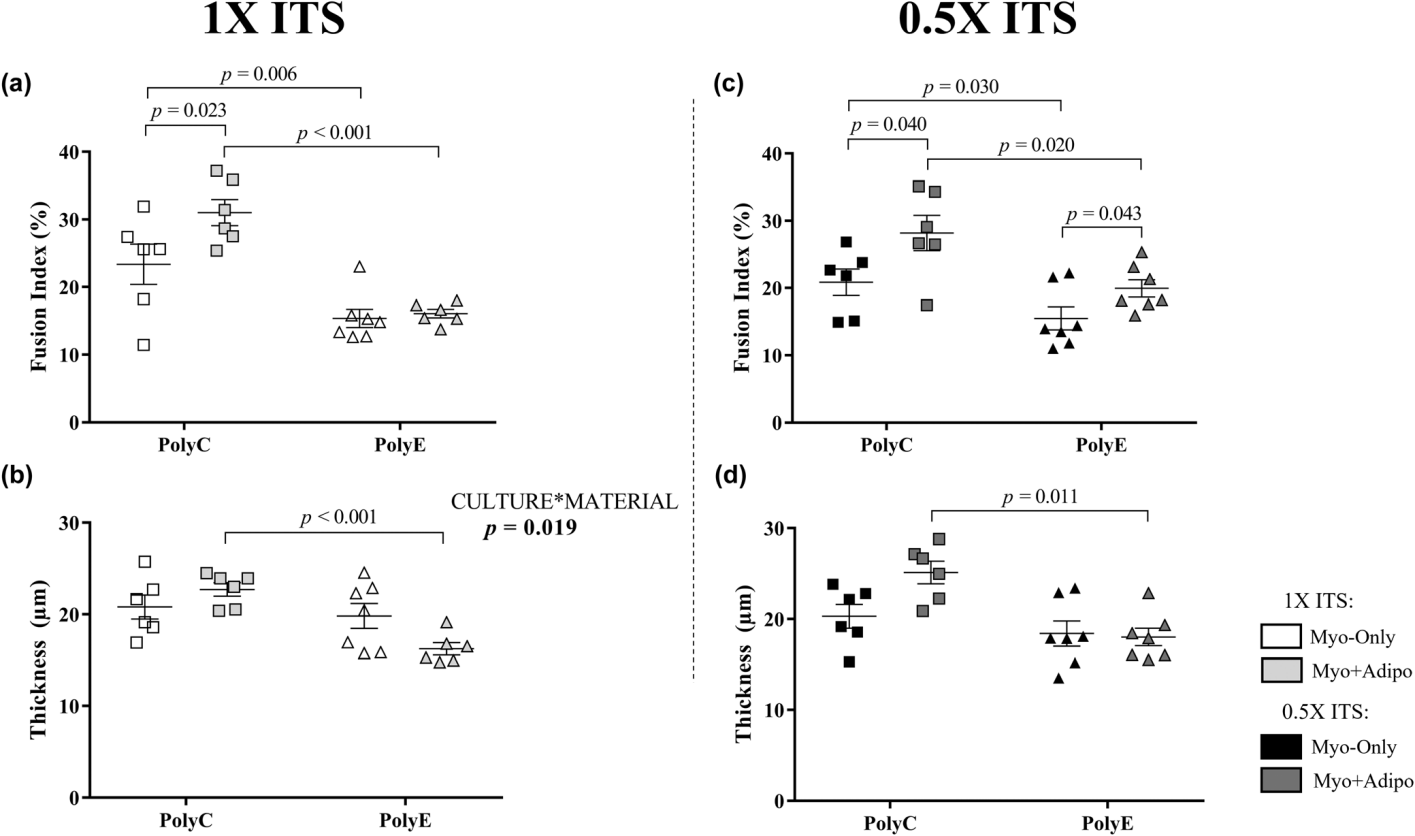
Fusion index and myotube thickness shown according to ITS concentrations: 1× ITS (a, b) and 0.5× ITS (c, d). Two‐way ANOVA (log‐*transformed* data) was used to assess the effect of co‐culture (CULTURE ‐ Myo‐Only vs. Myo + Adipo) and transwell membrane material (MATERIAL – PolyC vs. PolyE) within each ITS concentration. Fusion index is expressed as percentage (%), and myotube thickness as micrometers (μm). Data are represented as means ± SEM. Technical replicates: *n* = 6 for PolyC and *n* = 7 for PolyE. ITS, insulin‐transferrin‐selenium; PolyC, polycarbonate; PolyE, polyester.

At 1× ITS, the fusion index was higher with PolyC than PolyE membranes (MATERIAL, *p* < 0.001; Figure 4a). Figure 4a also shows an effect of CULTURE (*p* = 0.035); in the PolyC only, co‐culture (Myo + Adipo) increased (*p* = 0.023) fusion index vs. Myo‐Only. With regards to myotube thickness (Figure 4b), a CULTURE*MATERIAL interaction was observed (*p* = 0.019), indicating that the effects of culture depend on the type of transwell membrane. There was also a main effect of MATERIAL (*p* = 0.003) on myotube thickness (Figure 4b); in Myo + Adipo, cells cultured with PolyC had thicker myotubes (*p* < 0.001) than PolyE.

Similar to 1× ITS, cells cultured at 0.5× ITS had a higher fusion index with PolyC compared to PolyE (MATERIAL, *p* = 0.002; Figure 4c). There was also an effect of CULTURE (*p* = 0.006) at 0.5× ITS; in both PolyC and PolyE, there was a greater (*p* < 0.05) fusion index in Myo + Adipo vs. Myo‐Only (Figure 4c). While not significant, a trend (*p* = 0.08) consistent with the 1× ITS conditions was observed for CULTURE*MATERIAL in myotube thickness (Figure 4d), suggesting that the influence of culture may be modulated by the transwell membrane material. However, the effect of MATERIAL was significant (*p* = 0.002) for myotube thickness (Figure 4d). Consistent with the 1× ITS condition, Myo + Adipo co‐cultured cells had thicker myotubes (*p* < 0.001) with PolyC than PolyE.

### 3.3 Leptin and adiponectin diffusion vary across membrane materials

As leptin and adiponectin are exclusively secreted by adipocytes, we assessed the extent of diffusion of adipocyte‐derived factors from the lower to the upper compartment and evaluated how membrane material influences this process at both ITS concentrations. In Myo‐Only cultures, leptin and adiponectin remained below detection limits, confirming that upper‐compartment adipokines in co‐culture (Myo + Adipo) result from diffusion across the transwell membrane.

**FIGURE 5:**
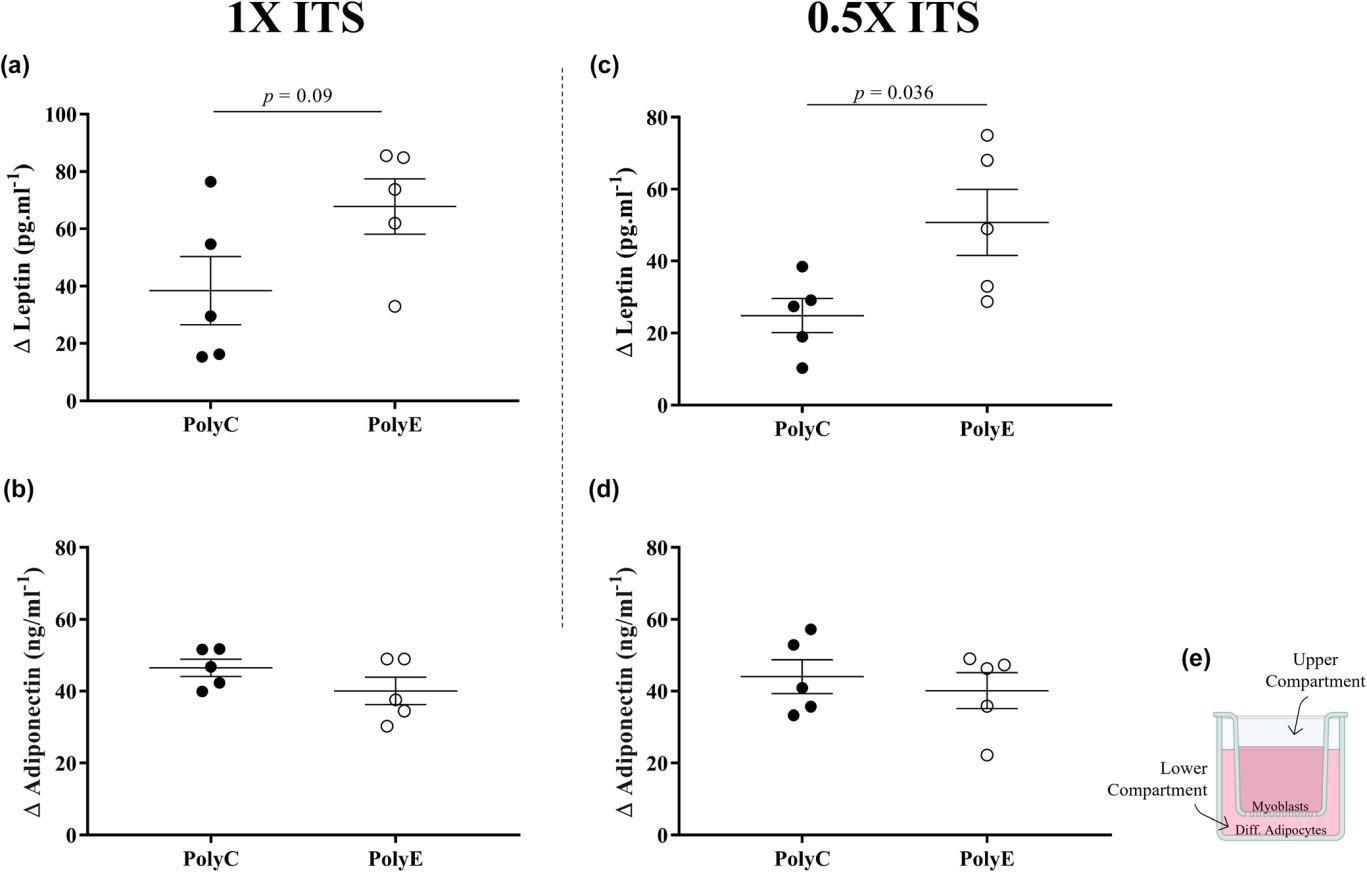
Delta leptin and adiponectin between co‐culture compartments shown according to ITS concentrations: 1× ITS (a, b) and 0.5× ITS (c, d). Unpaired t‐test was used to assess the effect of transwell membranes material (MATERIAL – PolyC vs. PolyE) within each ITS concentration. Delta leptin is expressed in pg.mL^−1^ (a, c) and adiponectin in ng.mL^−1^ (b, d) and was calculated as the difference between the lower (Diff. Adipocytes) and upper (Myoblasts) compartments of the co‐culture system (e). Data are represented as mean ± SEM (*n* = 5 for all conditions). ITS, insulin‐transferrin‐selenium, Diff. Adipocytes, differentiated adipocytes; PolyC, polycarbonate; PolyE, polyester.

At 1× ITS, the delta between compartments in leptin concentrations tended (*p* = 0.091) to be greater in PolyE (67.8 ± 9.7 pg.mL^−1^) than PolyC (38.4 ± 11.9 pg.mL^−1^; Figure 5a). This difference becomes significant (*p* = 0.036) at 0.5× ITS, where the difference between compartments in PolyE (50.8 ± 9.18 pg.mL^−1^) was twice that of PolyC (24.9 ± 4.79 pg.mL^−1^; Figure 5c), indicating that leptin diffusion across compartments is especially reduced in polyester membranes. Adiponectin diffusion remained similar across membranes at both 1× (*p* = 0.19; Figure 5b) and 0.5× ITS (*p* = 0.59; Figure 5d).

## 4 DISCUSSION

This study is the first to determine the effects of membrane material on primary human myoblast‐adipocyte transwell co‐culture systems and to reveal whether a common medium could be used for this system. By explicitly comparing polycarbonate and polyester membranes under standardized culture conditions, we provide direct evidence that membrane material significantly impacts myogenic outcomes. Compared to polyester membranes, myoblasts cultured on polycarbonate membranes exhibited a consistently greater fusion index, especially when they were co‐cultured with adipocytes, which also enhanced myotube thickness. We also found that both 0.5× and 1× ITS produced similar results for fusion index and thickness. Our results underscore the importance of membrane selection when using transwell systems for in vitro examination of adipocyte‐myoblast communication.

Our findings demonstrate that membrane material exerts a dominant influence on myogenic outcomes in transwell co‐culture systems. Although both membrane types share similar thickness (~10 μm) and pore size (0.4 μm), they differ markedly in pore density. Polycarbonate membranes used for our experiment have approximately 25‐fold more pores (~1 × 10^8^ pores/cm^2^) than polyester (~4 × 10^6^ pores/cm^2^) (Corning, 2014), likely enhancing the diffusion of adipocyte‐derived factors and promoting superior myotube differentiation across ITS concentrations. Increased porosity often correlates with softer membranes, and polycarbonate inserts are indeed more porous and mechanically softer than polyester. This softness, combined with higher porosity, creates a more compliant environment that supports myoblast differentiation (Kankala et al., 2019; Nunes‐Pereira et al., 2015; van Santen et al., 2022). Although polyester is often selected for better microscopy visualization, its inconsistent performance in our study seems to outweigh this advantage (Chung et al., 2018). Furthermore, variations in pore density across membrane materials complicate direct comparisons based solely on material composition.

In the myoblast‐adipocyte co‐culture, the differences we observed in fusion index increased from ~16% to 19% with the polyester membrane to ~28%–31% in the polycarbonate membrane. Though the baseline fusion index was modest, there was a 9 to 15‐point jump in fusion index between the membranes, reflecting a ~50% relative increase in the 0.5× ITS and a ~95% relative increase in the 1× ITS conditions. This jump is statistically significant, consistently observed, and reflects a meaningful biological improvement in differentiation conditions beyond experimental noise. The improvements in differentiation conditions with the polycarbonate membrane are further supported by a corresponding relative increase of 40% in myotube thickness. Taken together, these findings clearly show that polycarbonate membranes provide a substantially more supportive environment for myogenic differentiation compared to polyester membranes.

Adiponectin and leptin were measured in each compartment as representative factors secreted exclusively by adipocytes. The examination of these adipokines allowed us to assess the extent to which adipocyte‐derived factors from the lower compartment diffuse into the upper compartment. At both ITS concentrations, membrane material resulted in greater leptin diffusion with the polycarbonate than with the polyester membrane. The small molecular size of leptin (~16 kDa) (Ahima & Flier, 2026) likely facilitated its passage through the higher pore density of the polycarbonate membrane. In animal models, leptin (100–1000 ng/mL) has been shown to impair differentiation in rodent (C2C12) and porcine myoblasts through JAK/STAT3 and MEK/ERK signaling (Pijet et al., 2013; Yu et al., 2008). However, the direct effects of leptin on human myoblast differentiation remain largely unexplored in vitro (Solberg et al., 2005). In our study, despite greater leptin diffusion with the polycarbonate membrane, the differentiation of myoblasts was not impaired. This lack of an effect of leptin in the upper compartment may be explained by lower leptin levels than that reported to affect myoblast differentiation in vitro, which are usually reached by adding leptin directly to the culture medium (Pijet et al., 2013; Solberg et al., 2005; Yu et al., 2008).

In contrast, adiponectin diffusion was not affected by membrane material, as delta adiponectin did not differ between polycarbonate and polyester at either ITS concentrations. Unlike leptin, adiponectin assembles into high‐molecular‐weight multimers (~300 kDa) (Fang & Judd, 2018; Zhao et al., 2021), which likely encounter size constraints regardless of membrane material. This may explain why diffusion was similarly limited across both polycarbonate and polyester membranes despite differences in pore density. The effects of adiponectin on myogenesis are context‐dependent and vary by cell type and species: globular adiponectin promotes myogenic differentiation and fusion in C2C12 myoblasts (Fiaschi et al., 2009), while inhibiting myogenic differentiation in avian satellite cells (Guo et al., 2025). In human primary myoblasts, direct effects of adiponectin on differentiation remain largely unexplored, although adiponectin signaling through AdipoR1‐AMPK seems to support myogenic capacity and mitochondrial function (Sente et al., 2016).

These findings expose a critical gap in the field. Most studies employing transwell co‐culture systems to investigate skeletal muscle‐adipose tissue interactions do not report the membrane material used. While pore size and manufacturer are often mentioned, membrane composition, including reference to the product, is frequently omitted (Bruckbauer & Zemel, 2011; Choi & Myung, 2014; Dietze et al., 2002; Kudoh et al., 2018; Ojima et al., 2025; Pandurangan et al., 2015; Park et al., 2013; Seo et al., 2019; Sun & Zemel, 2009) limiting reproducibility and cross‐study comparisons. Our results emphasize the importance of reporting membrane type and pore density to improve methodological transparency and rigor in future research studies.

Beyond membrane selection, both 0.5× and 1× ITS supplementation supported comparable differentiation outcomes and adipokine secretion. Though the 1× ITS produced similar results, given that myoblasts are typically differentiated in the absence of ITS and adipocytes require 1× ITS, the 0.5× ITS represents a compromised level that provides sufficient ITS to maintain adipocyte function and adipokine secretion while avoiding the direct effects that ITS could have on myoblast differentiation. Given that myogenic differentiation was comparable between 0.5× and 1× ITS conditions, the use of 0.5× ITS may minimize confounding effects of ITS while preserving the integrity of adipocyte‐myocyte crosstalk. This approach aligns with previous reports of successful intermediate media strategies in other co‐culture systems (Anvari & Bellas, 2021; Besser et al., 2020; Vis et al., 2020), suggesting that balanced formulations can support dual cell types effectively.

A key strength of this study is the use of primary human cells, including preadipocytes isolated from abdominal subcutaneous adipose tissue and primary human myoblasts. The use of primary human‐derived preadipocyte and myoblast cells in co‐culture models is rare in the literature and represents a unique approach. This rarity may be because of the complexity and challenges associated with obtaining and culturing primary human cells. The use of these cells enhances the physiological relevance of the co‐culture system, beyond what can be achieved with the immortalized or non‐human‐derived cell lines that are mostly used in other studies (Choi & Myung, 2014; Kudoh et al., 2018; Park et al., 2013; Seo et al., 2019; Sun & Zemel, 2009). The adipocyte differentiation protocol employed is a robust and well‐validated multistage approach, ensuring mature adipocytes with stable adipokine secretion profiles at the time of co‐culture. The transwell‐based system allowed us to isolate the paracrine effects of adipocytes on myogenic differentiation while keeping cell populations physically separated, providing a controlled environment to compare the biological impact of membrane material and ITS concentration. Additionally, the measurement of adipokines in each chamber provides insight into experimental conditions that affected our cellular outcomes. Finally, the inclusion of multiple technical replicates and the systematic comparison of two widely used but rarely compared membrane materials (polycarbonate and polyester) offers valuable methodological insight for researchers employing transwell co‐culture systems.

Despite these strengths, several limitations should be considered when interpreting the findings. First, we did not examine the physicochemical properties of the membranes in detail because this line of inquiry was outside the scope of our objective, which was to evaluate how membrane material and ITS concentration affect the differentiation of myoblasts in adipocyte co‐culture. Future studies should directly characterize the effects of the physicochemical properties of the membrane materials themselves (e.g., surface chemistry, topography, or mechanical properties) so to attribute the observed effects to specific structural features beyond the known differences in pore density. In particular, the 0.4 μm pore size used in this study may limit the diffusion of larger adipocyte‐derived factors, suggesting that increasing the pore size could enhance paracrine communication in future co‐culture experiments. Though the use of cells from a single donor limits generalizability, doing so ensured genetic consistency and minimized variability in our outcomes, allowing us to focus on delineating the effects of membrane material and ITS concentrations in the media. The inserts were coated in Matrigel to standardize myoblast attachment and differentiation (Barbero et al., 2001; Josan et al., 2021). This coating could have masked differences between membranes. However, despite the presence of Matrigel, distinct effects of membrane material and culture conditions were observed, highlighting the robustness of the findings. Despite these limitations, the results provide a foundation for refining co‐culture strategies in future experiments. To minimize variability between manufacturers, only Corning® membranes were tested. Further studies are needed to determine whether our results could be replicated with other commercially available inserts.

In conclusion, membrane material is a critical determinant of myogenic outcomes and factors affecting crosstalk in skeletal muscle‐adipose tissue co‐culture systems. Although all inserts were coated with matrigel to standardize attachment, differences persisted, suggesting that these effects are driven by membrane properties. Polycarbonate membranes, with their higher pore density and softer structure, likely enhance the secretion and diffusion of adipocyte‐derived factors, creating a more compliant environment that supports myoblast differentiation. In addition, the 0.5× ITS‐based medium appears to maintain adipocyte function while promoting myogenesis. Together, these findings underscore the importance of reporting membrane characteristics and optimizing media composition to improve reproducibility and interpretation of results, thereby advancing in vitro models of skeletal muscle‐adipose tissue interactions.

## AUTHOR CONTRIBUTIONS

**Claire Plissonneau:** Conceptualization; data curation; formal analysis; investigation; methodology; validation; visualization. **Bjorn T. Tam:** Conceptualization; data curation; formal analysis; investigation; methodology; supervision; validation; visualization. **Pierre Y. Garneau:** Data curation; formal analysis; validation. **José A. Morais:** Data curation; formal analysis; validation. **Alisa Piekny:** Data curation; formal analysis; methodology; validation. **Sylvia Santosa:** Conceptualization; data curation; formal analysis; funding acquisition; investigation; methodology; project administration; resources; supervision; validation; visualization.

## FUNDING INFORMATION

S.S. holds a Canadian Research (Tier 2) in Clinical Nutrition. C.P. is supported by a Horizon Postdoctoral Fellowship from Concordia University. This work was supported by a Discovery Grant from the Natural Sciences and Engineering Research Council of Canada (NSERC).

## CONFLICT OF INTEREST STATEMENT

The authors declare no conflicts of interest.

## Data Availability

The data sets generated during this study will be made available from the corresponding author upon reasonable request pending ethics approval.
